# Case Report: Combination of sirolimus and endoscopic lauromacrogol sclerotherapy in the management of blue rubber bleb nevus syndrome with gastric tract bleeding

**DOI:** 10.3389/fped.2024.1488466

**Published:** 2025-01-10

**Authors:** Lu Liu, Liyuan Wang, Fan Hu

**Affiliations:** ^1^ Department of Pediatric Cardiology, West China Second University Hospital, Sichuan University, Chengdu, Sichuan, China; ^2^ Key Laboratory of Birth Defect and Related Diseases of Women and Children (Sichuan University), Ministry of Education, Chengdu, Sichuan, China; ^3^ Department of Pediatric Ganstroenterology, West China Second University Hospital, Sichuan University, Chengdu, Sichuan, China

**Keywords:** blue rubber bleb nevus syndrome, sirolimus, venous malformation, lauromacrogol, sclerotherapy, endoscopy

## Abstract

**Background:**

Blue rubber bleb nevus syndrome (BRBNS) is a rare venous malformation disorder. Currently, there is no standard therapy for this disease. However, lauromacrogol, a sclerosant extensively utilized in the management of vascular malformations, has been applied in the treatment of BRBNS. Research on the combined therapy of sirolimus and lauromacrogol for the treatment of BRBNS remains limited.

**Case summary:**

Here, we report the case of a 12-year-old girl diagnosed with BRBNS. The patient presented with chronic anemia and skin “hemangioma.” The examinations showed severe anemia, along with decreased serum iron and ferritin levels. Magnetic resonance imaging showed abnormal nodular lesions in various parts of the intestine. The patient was treated with a combination of endoscopic sclerotherapy using lauromacrogol and oral sirolimus. After 1 year of treatment, the patient showed no signs of anemia or gastrointestinal tract bleeding.

**Conclusion:**

BRBNS is a rare disorder that is often misdiagnosed, especially by inexperienced pediatricians. The combination of oral sirolimus with endoscopic lauromacrogol has demonstrated efficacy in reducing lesion size and elevating hemoglobin levels.

## Introduction

Blue rubber bleb nevus syndrome (BRBNS) is a rare venous malformation disorder (1). It is characterized by multiple lesions in the skin and gastrointestinal (GI) tract, with occasional involvement of other organs such as the liver and central nervous system (2). The precise incidence of BRBNS remains unknown, with approximately 350 cases documented in the literature to date. The lesions, known as blebs, can vary in size and number, and they often cause symptoms such as bleeding, anemia, and, in severe cases, significant gastrointestinal hemorrhage.

Despite the incomplete understanding of the underlying mechanism of blue rubber bleb nevus syndrome, it is widely accepted that somatic mutations in the TEK gene (TEK receptor tyrosine kinase) play a crucial role in its etiology (3). These mutations give rise to irregular development and expansion of blood vessels and are associated with a range of vascular malformations. As our understanding of the role of TEK mutations in the development of these lesions deepens, it opens up new ways for targeted therapies that could potentially inhibit abnormal blood vessel growth and alleviate the associated clinical manifestations.

Owing to the limited number of BRBNS patients and the lack of extensive case studies on its treatment, empirical treatment remains the principal focus of reports in various centers. Commonly used management strategies for BRBNS with GI tract bleeding include oral sirolimus, endoscopic treatment, surgery, and combinations of these methods (4–7). Lauromacrogol is a widely used sclerosant for the treatment of vascular malformations. It functions by inducing thrombosis and subsequent fibrosis in blood vessels, which can help reduce bleeding and prevent the recurrence of lesions. There are limited studies on the use of lauromacrogol in treating BRBNS patients, especially in combination with sirolimus.

We hereby present a successful case of treating BRBNS with GI tract bleeding using a combination of sirolimus administration and endoscopic sclerotherapy.

## Case description

A 12-year-old Chinese girl presented to our hospital with a 2-year history of pale complexion and fatigue.

The symptoms started 2 years ago. She presented to a local hospital. A complete blood cell (CBC) count revealed decreased hemoglobin (Hb) levels of 58 g/L. After conducting a series of tests, including ferritin, serum iron, transferrin, and a bone marrow examination, she was diagnosed with iron deficiency anemia (IDA). She received blood transfusions and chalybeate treatment. Despite this, her CBC continued to indicate refractory anemia, and she received seven blood transfusions over the following 2 years. Gastroscopy and enteroscopy conducted at the local hospital showed multiple “hemangiomas” in the stomach, duodenum, and large intestine. Subsequently, she was referred to our hospital for further treatment.

At birth, she was found to have “hemangiomas” on her skin and lower lip. No diagnostic examinations or treatments were performed for the lesions. Her parents denied any family history of vascular anomalies and GI tract bleeding.

**Figure 1:**
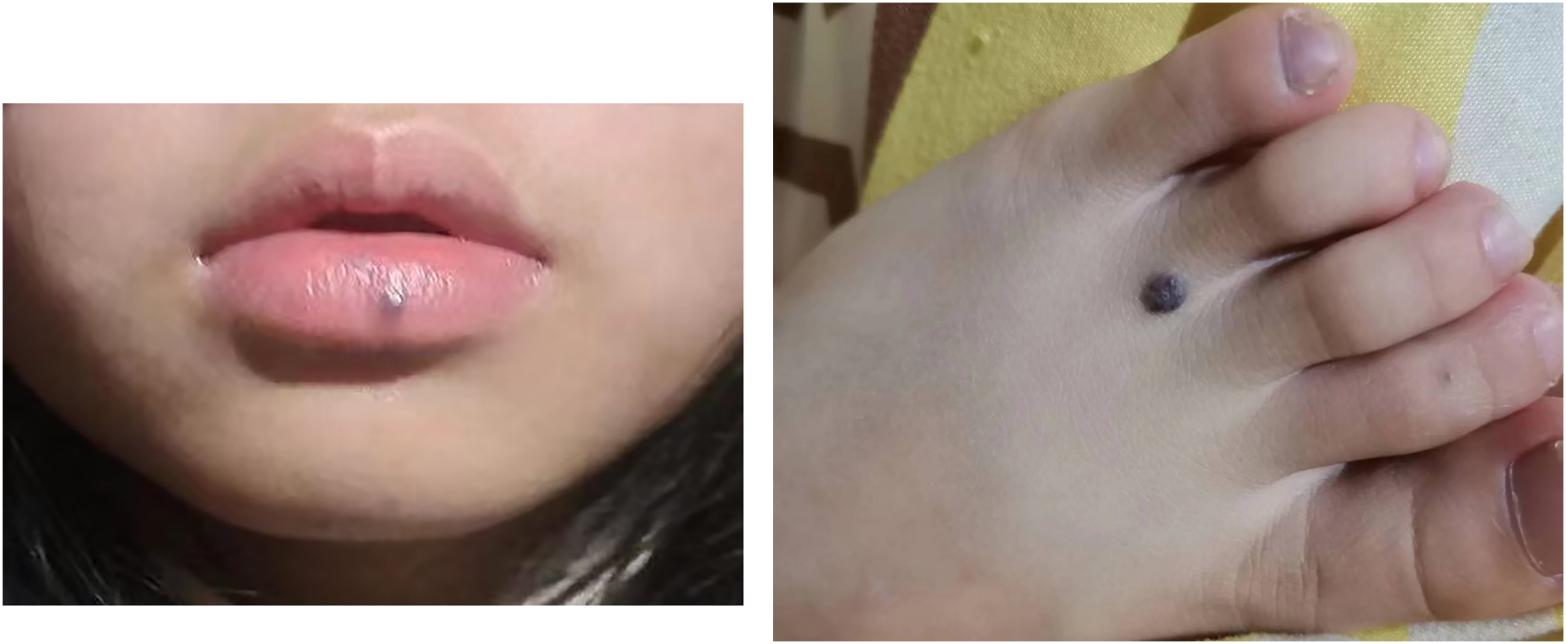
Lesions on the lip and foot of the patient.

Her vital signs were as follows: body temperature, 36.8℃; heart rate, 105 beats per minute; respiratory rate, 22 beats per minute; and blood pressure, 114/59 mmHg. She appeared pale. Purple-black or purple-blue nodular lesions were observed on her lower lip and skin, including the neck, upper limbs, and lower limbs (Figure 1). The size of these lesions ranged from 0.5 to 2 cm.

**Figure 2:**
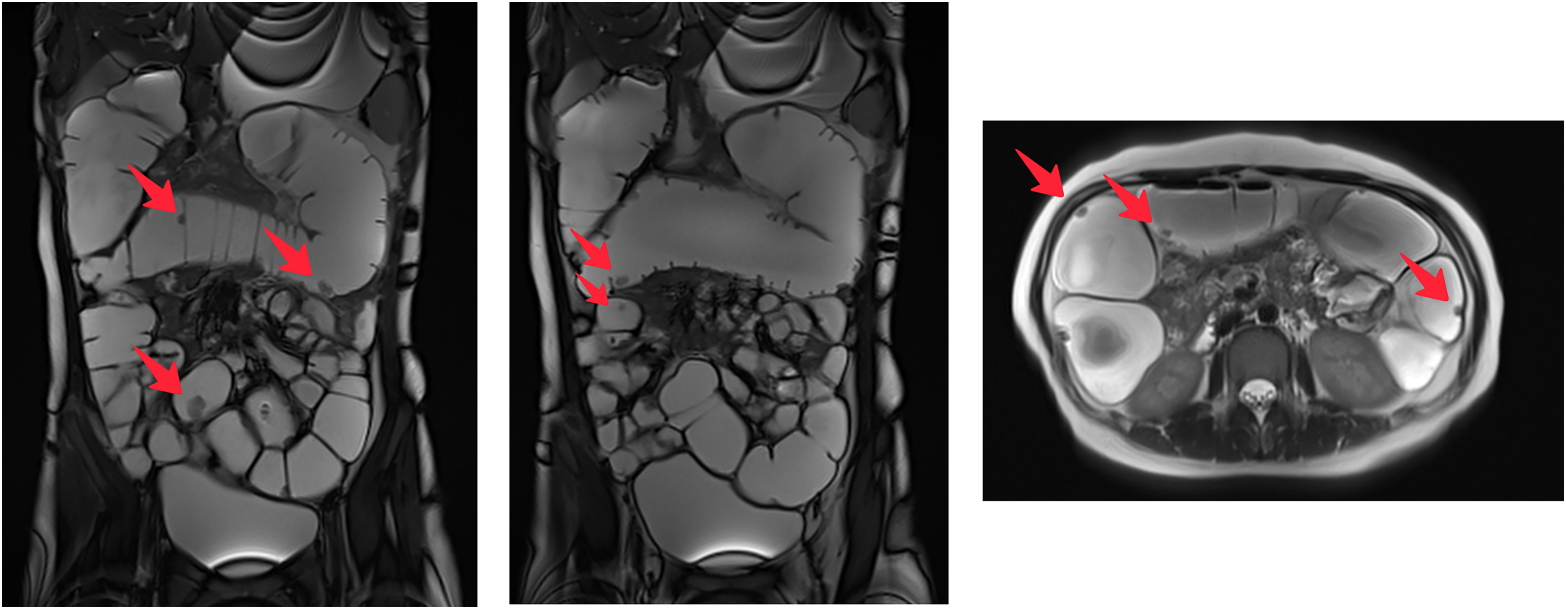
Magnetic resonance imaging showing abnormal signals at various sections of the intestine.

The complete blood cell count revealed decreased red blood cells (RBCs) at 2.34 × 10^12^/L and low Hb at 48 g/L. The platelet count was normal. Coagulation function test results were as follows: prothrombin time, 11.9 s; activated partial thromboplastin time, 21.4 s; fibrinogen, 213 mg/dl; and thrombin time, 17 s. Iron deficiency was established with a serum iron level of 1.2 µmol/L, transferrin level of 3.38 g/L, and serum ferritin level of 2.30 ng/ml. The fecal occult blood test was positive. Abdominal magnetic resonance imaging showed multiple enhanced nodules, measuring about 0.5–1.5 cm, with high T2 weighted imaging (T2WI) signals in the small intestine, colon, and rectum (Figure 2).

**Figure 3:**
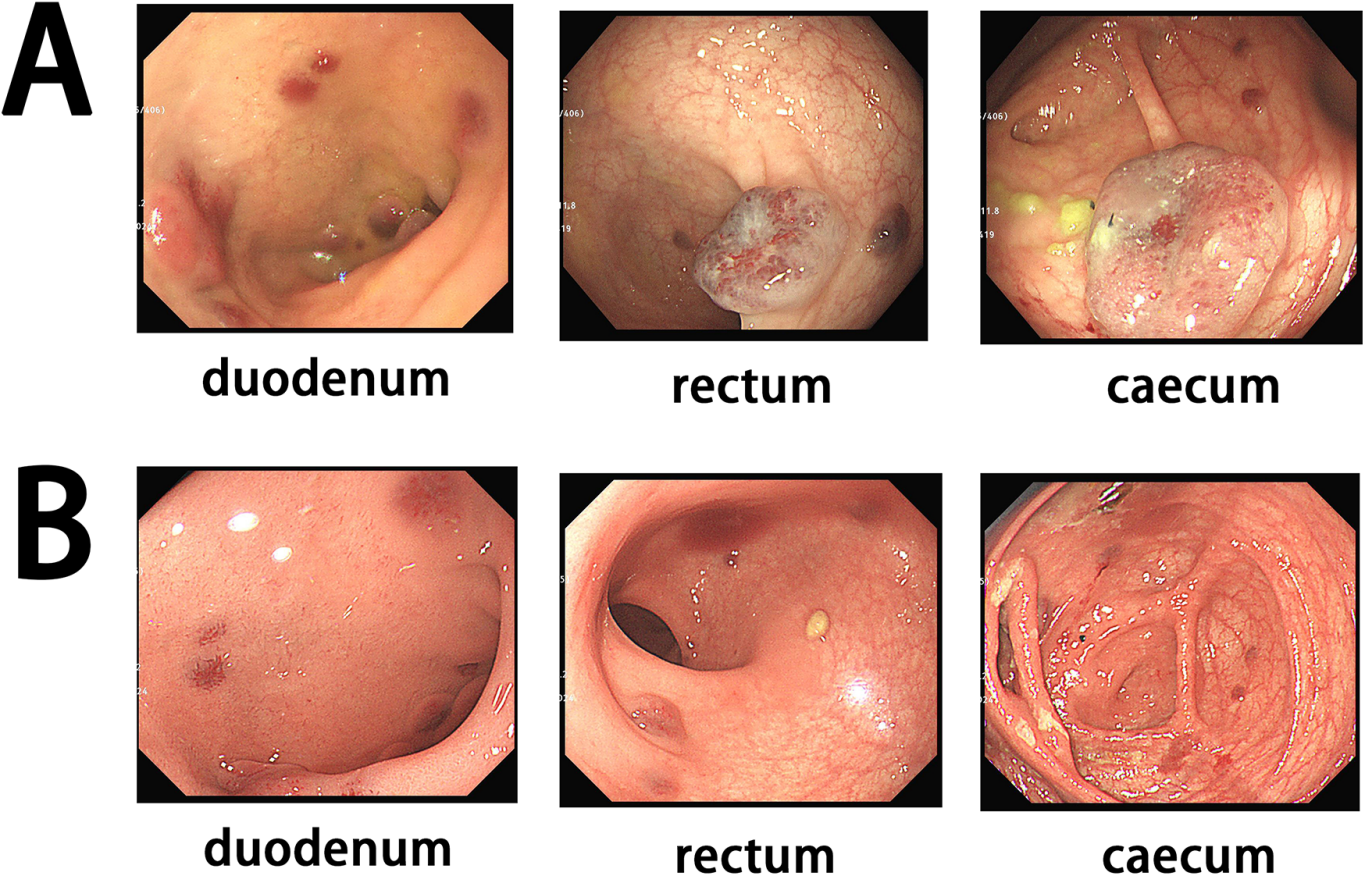
**(A)** Initial endoscopy showing multiple lesions. **(B)** Endoscopy after treatment suggesting a significant decrease of the lesions.

Based on the clinical manifestations and enteroscopic findings, a diagnosis of BRBNS was established. The patient was treated with one blood transfusion and oral chalybeate. The patient underwent gastroscopy and enteroscopy, which revealed multiple vascular nodules (Figure 3A). After that, lauromacrogol foam was injected into the lesions measuring approximately ≥1 cm in diameter (Figure 3B). The foam was prepared by mixing lauromacrogol with air (1:4). After that, oral sirolimus was initiated at a starting dose of 1.6 mg/m^2^/day. The dosage was adjusted based on the trough concentration. Initially, the target trough concentration was 10–15 ng/ml. Once the blood Hb level of the patient normalized, the target trough concentration was reduced to 5–10 ng/ml. The Hb level improved to 89 g/L, and the patient was discharged.

After 3 months, the patient underwent follow-up gastroscopy and enteroscopy, which showed a significant decrease in the size of the lesions (Figure 3). Her Hb level had increased to 140 g/L, and the fecal occult blood test was negative. After 1 year, the patient showed no signs of anemia or GI tract bleeding, with an Hb level of 132 g/L.

## Discussion

BRBNS, a congenital vascular disease, can be diagnosed at any age. Although the occurrence of gastrointestinal tract bleeding is relatively frequent in this disease, its rarity often leads to misdiagnosis and delayed treatment. Small intestine lesions are most commonly seen in GI tract involvement (8). Chronic gastrointestinal tract bleeding may lead to IDA. In this case, the patient presented with refractory IDA and was managed with blood transfusions and chalybeate therapy over several years. Similar instances of delayed diagnosis have also been reported in previous studies (9).

Currently, there is no standardized management for BRBNS with GI tract bleeding. Double (*cis*) mutations of the TEK gene were observed in BRBNS (3). The TEK gene encodes tyrosine kinase with immunoglobulin and epidermal growth factor homology domains 2 (TIE2), which activates the phosphatidylinositol-3-kinase (PI3K)/mammalian target of rapamycin (mTOR) pathway and participates in vascular growth (10). Sirolimus, an mTOR inhibitor, is capable of blocking the TIE2/PI3K/mTOR pathway, thereby exerting inhibitory effects on vascular lesions (11). A prospective study was conducted involving 11 patients treated with sirolimus. The results indicated a significant increase in hemoglobin levels, with only one patient having received a single blood transfusion (12). The optimal dosage and duration of sirolimus therapy for BRBNS remain undetermined. A trough concentration ranging from 3 to 10 ng/mL has exhibited favorable results (12). Even lower concentration ranges were reported, but a persistent occurrence of gastrointestinal bleeding was observed (13). Available evidence indicates that sirolimus shows inhibitory effects on GI tract bleeding and lesion growth, yet it fails to achieve a complete cure of the underlying disease. In reported cases, patients are required to maintain long-term administration of the drug. In this case, the initial target trough concentration was set at 10–15 ng/ml to ensure prompt management of gastrointestinal tract bleeding. Subsequently, the concentration was adjusted to 5–10 ng/ml for maintenance.

Endoscopy plays a crucial role in the diagnosis and treatment of BRBNS. Capsule endoscopy can reveal the overall lesions present in the GI tract (5). However, both gastroscopy and enteroscopy are employed for diagnostic and therapeutic purposes. Previous studies have included endoscopic sclerotherapy and resection as potential treatment options (5). To date, no studies have compared the results of these two methods. Lauromacrogol, as a sclerosant, has been used in the treatment of gastric varices and venous malformations (14, 15). In this case, sclerotherapy was injected into large lesions via endoscopy, leading to a significant reduction in size. The combination of sirolimus and sclerotherapy has the potential to effectively decrease the lesions in the GI tract.

Aggressive surgical resection to remove all GI tract lesions was a common approach before the era of sirolimus (16). However, in recent years, surgical resection has usually been performed at lesion-concentrated segments (8). In this case, the GI tract lesions were diffusely distributed, particularly in the small intestine. After carefully considering the patient age and the need to minimize tissue damage, we opted for sclerotherapy as the preferred treatment instead of surgical resection.

## Conclusion

BRBNS is a rare condition that frequently results in misdiagnosis, particularly by less experienced pediatricians. The presence of typical skin lesions alongside refractory IDA is a critical sign for further endoscopic examination. Treatment options should be tailored to the unique characteristics of each patient. The combination of oral sirolimus and endoscopic lauromacrogol has been shown to be effective in reducing lesion size and increasing hemoglobin levels.

## Data Availability

The original contributions presented in the study are included in the article/Supplementary Material, further inquiries can be directed to the corresponding author.
